# Techno-economic analysis and climate change impacts of sugarcane biorefineries considering different time horizons

**DOI:** 10.1186/s13068-017-0722-3

**Published:** 2017-03-14

**Authors:** Tassia L. Junqueira, Mateus F. Chagas, Vera L. R. Gouveia, Mylene C. A. F. Rezende, Marcos D. B. Watanabe, Charles D. F. Jesus, Otavio Cavalett, Artur Y. Milanez, Antonio Bonomi

**Affiliations:** 1 Laboratório Nacional de Ciência e Tecnologia do Bioetanol (CTBE), Centro Nacional de Pesquisa em Energia e Materiais (CNPEM), Caixa Postal 6192, Campinas, SP CEP 13083-970 Brazil; 2 Faculdade de Engenharia Química, Universidade Estadual de Campinas (UNICAMP), Campinas, SP Brazil; 3 Departamento de Biocombustíveis, Banco Nacional de Desenvolvimento Econômico e Social (BNDES), Rio de Janeiro, RJ Brazil

**Keywords:** Ethanol, Sugarcane, Energy cane, Production costs, Climate change

## Abstract

**Background:**

Ethanol production from lignocellulosic feedstocks (also known as 2nd generation or 2G ethanol process) presents a great potential for reducing both ethanol production costs and climate change impacts since agricultural residues and dedicated energy crops are used as feedstock. This study aimed at the quantification of the economic and environmental impacts considering the current and future scenarios of sugarcane biorefineries taking into account not only the improvements of the industrial process but also of biomass production systems. Technology assumptions and scenarios setup were supported by main companies and stakeholders, involved in the lignocellulosic ethanol production chain from Brazil and abroad. For instance, scenarios considered higher efficiencies and lower residence times for pretreatment, enzymatic hydrolysis, and fermentation (including pentoses fermentation); higher sugarcane yields; and introduction of energy cane (a high fiber variety of cane).

**Results:**

Ethanol production costs were estimated for different time horizons. In the short term, 2G ethanol presents higher costs compared to 1st generation (1G) ethanol. However, in the long term, 2G ethanol is more competitive, presenting remarkable lower production cost than 1G ethanol, even considering some uncertainties regarding technology and market aspects. In addition, environmental assessment showed that both 1G (in the medium and long term) and 2G ethanol can reduce climate change impacts by more than 80% when compared to gasoline.

**Conclusions:**

This work showed the great potential of 2G ethanol production in terms of economic and environmental aspects. These results can support new research programs and public policies designed to stimulate both production and consumption of 2G ethanol in Brazil, accelerating the path along the learning curve. Some examples of mechanisms include: incentives to the establishment of local equipment and enzyme suppliers; and specific funding programs for the development and use of energy cane.

**Electronic supplementary material:**

The online version of this article (doi:10.1186/s13068-017-0722-3) contains supplementary material, which is available to authorized users.

## Background

Replacing fossil fuels by renewable alternatives to reduce dependence on fossil resources and greenhouse gas (GHG) emissions has received special attention worldwide in the last decades. In Brazil, energy consumption in the transportation sector has increased by 65% in the past decade, reaching 86.3 million metric tons of oil equivalent (Mtoe) in 2014, with gasoline and ethanol representing 30 and 15% of this total, respectively [1]. In the Brazilian transportation sector, hydrous ethanol is used in the flex-fuel vehicles and anhydrous ethanol is mixed to the gasoline (18–27.5% v/v) for use in the gasoline-powered vehicles [2].

Ethanol is conventionally produced through first-generation (1G) process, based on the conversion of extractable sugars and starch (mostly from sugarcane and corn, respectively). The 1G ethanol production from sugarcane in Brazil is a consolidated large-scale process. This experience is based on a 40-year experience motivated by the creation of PROALCOOL program in the 1970s. The learning curve of 1G sugarcane ethanol has shown that significant reductions in production cost were achieved over the years, due to the gains in agricultural and industrial yields and to the increase in the scale of production [3, 4].

Ethanol can also be produced through second-generation (2G) process using lignocellulosic materials, such as agricultural residues and dedicated energy crops, as feedstock [5–7]. In Brazil, parts of sugarcane lignocellulosic fractions (bagasse and straw) are identified as main feedstocks for 2G ethanol production [8], taking advantage of the possible industrial 1G and 2G integration by sharing infrastructure and increasing potential for energy optimization, among other benefits [9, 10].

Even though 2G ethanol has reached commercial scale with a few plants installed worldwide, including two commercial plants in Brazil that recently started operation (2014/2015), this process is still at the beginning of its technological learning curve [11]. Some studies suggest that production cost of 2G ethanol is still higher than that of 1G ethanol, due to the higher capital expenditures and operating expenses [9, 12, 13]. Therefore, at this initial stage it is likely that 2G ethanol will depend on governmental polices and incentives, such as the RFS (Renewable Fuel Standard) in USA [14, 15]. In Brazil, special credit lines for research and development (R&D) on biomass conversion and for the construction of 2G ethanol plants were available within the PAISS initiative—joint plan for supporting industrial technological innovation in the sugar-based energy and chemical sectors [16]. While in Brazil most incentives focus on providing funds for R&D and plant implementation, in other countries, especially in the USA, mechanisms to increase the consumption of 2G ethanol have been practiced [17].

Techno-economic and environmental assessments have been increasingly used to compare different process configurations for 2G ethanol production, including combination of pretreatments, variation in enzyme dosages, alternative pentoses utilization, among others [18–20]. Evaluation of sugarcane biorefineries considering target yields of 2G process showed that an integrated 1G2G plant can be more profitable than a 1G plant. Also, utilization of pentoses for ethanol production instead of biodigestion is an important driver for the reduction in production costs [9, 21]. In terms of environmental impacts, Dias et al. [9] showed that high consumption of chemicals in 2G process, e.g., in the delignification step, can increase climate change impacts compared to 1G ethanol production process.

Some other studies have evaluated expected advances for integrated 1G2G process in Brazil. Silva et al. [22] carried out a life cycle assessment (LCA) of prospective 1G and 1G2G scenarios (2020–2030) compared to current ethanol production. Jonker et al. [23] evaluated economic results for different biomass crops and industrial technologies considering 2010 and 2030 scenarios. This study showed that ethanol production costs decrease over time due to increase of industrial scale, biomass yield, and industrial efficiency. Wang et al. [13] performed an economic and GHG emission analysis of sugarcane ethanol production considering the projections for 2010–2020 period, with most parameters based on literature. Results indicated that the combined production cost of 1G and 2G ethanol can be significantly reduced over time, being comparable to 1G cost in 2020. A similar trend was observed for climate change impacts.

However, these studies projecting 2G technologies were based on literature and without a set of scenarios representing a continuous learning curve for both 1G and 2G ethanol production processes. In this work, production costs and climate change impacts for 1G and 2G ethanol were quantified considering the technological and economic projections between 2015 and 2030. The projections considered improvements not only of industrial processes but also of biomass production systems (e.g., inclusion of energy cane as feedstock). It is important to highlight that these scenarios were set up and discussed with specialists from main companies and stakeholders, from Brazil and abroad, involved in the lignocellulosic ethanol production chain. The objective was to forecast the technological learning curve for both 1G and 2G ethanol production processes, including both biomass production and industrial conversion, and to quantify economic and environmental impacts of present and future technologies for ethanol production. These results can support the proposition of research programs and public policies to stimulate both production and consumption of 2G ethanol in Brazil.

## Methods

The Virtual Sugarcane Biorefinery (VSB), developed at the Brazilian Bioethanol Science and Technology Laboratory (CTBE), was employed to perform the techno-economic and environmental assessment of sugarcane biorefineries. The VSB is a computational framework that allows simulation and evaluation of the entire sugarcane chain and other biomasses (from biomass production to final products use) considering the three pillars of sustainability: economic, environmental, and social [24]. In this work, comparison between 1G plants and integrated 1G and 2G (1G2G) ethanol production units was carried out, considering three time horizons: short (2016–2020), medium (2021–2025), and long (2026–2030) terms.

### Scenarios definition

Unlike most industrial facilities, sugarcane biorefineries operate only about 6–8 months per year, since it is limited by the sugarcane harvesting period and, in some areas, by the raining season. As a result, costs related to investment in equipment have significant contribution towards ethanol production cost. Therefore, it is important to take into account strategies to extend operational period in the future of these biorefineries. In this context, energy cane, a sugarcane variety with higher fiber content, is seen as a promising alternative not only because of its high biomass yields but also due to its potential to extend both 1G and 2G ethanol production and electricity generation periods.

Scenarios definition considered improvements of sugarcane and energy cane production systems, industrial conversion (both 1G and 2G processes), and market perspectives (enzyme cost and investment in equipment) according to the three defined time horizons. As mentioned before, these assumptions and scenarios setup were supported by main companies, stakeholders, and specialists from Brazil and abroad who were involved in the lignocellulosic ethanol production chain.

For 1G process, a base case was selected as the benchmark to represent the “average” existing autonomous distilleries in Brazil. It is an industrial plant processing 2 million metric tons of sugarcane per year, producing only ethanol as output. Due to the energy-intensive process and inefficient low-pressure boilers, this plant does not export electricity to the grid but is self-sufficient in energy terms. The other scenarios considered a modern autonomous distillery processing 4 million metric tons of sugarcane per year, recovering straw from the fields and, in the medium term onwards, using energy cane as additional feedstock. In this configuration, thermal integration and high-pressure systems for cogeneration of heat and power (CHP) allow not only being self-sufficient in energy terms but also export surplus electricity to the grid.

**Fig. 1:**
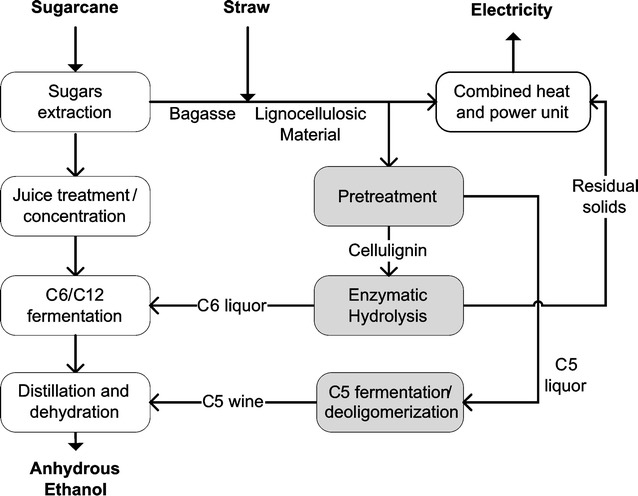
Block flow diagram for integrated 1G2G process. Process steps usually found in 1G autonomous distilleries (*white* blocks) and additional steps relative to 2G process (*light gray* blocks)

The configuration of 2G process was based on steam explosion pretreatment, pentoses (C5 sugars) liquor separation, enzymatic hydrolysis, fermentation of hexoses (C6 sugars) along with 1G juice. Fermentation of pentoses is carried out separately from C6 and 1G juice. After fermentation, both alcoholic streams are sent to a series of distillation columns and dehydration processes where anhydrous ethanol (99.6 wt%) is obtained. Lignin-rich residual solids from enzymatic hydrolysis are burnt in the CHP as supplementary fuel. Figure 1 shows a representation of the integrated 1G2G process and Table 1 summarizes the main assumptions for the evaluated scenarios. Detailed information about biomass composition and production system (productivities, mechanization level, others) as well as industrial process (e.g., operational conditions and yields) according to the technology levels and timeframes are presented in the Additional file 1.

**Table 1:** Main characteristics of the evaluated scenarios

Scenarios^a^	1G-base	1G-ST	1G2G-ST	1G-MT	1G2G-MT	1G-LT	1G2G-LT
Sugarcane processing (10^6^ t/year)^b^	2	4	4	4	4	4	4
Straw recovery (%)^c^	–	50	50	60	60	70	70
Energy cane processing (10^6^ t/year)	–	–	–	1.72^d^		4.17^e^	
1G technology level	Base	Optimized (high-pressure boilers and reduced steam consumption)					
2G yield (L/t LCM)^f^	–	–	~240	–	~295	–	~350
Vinasse biodigestion efficiency (%)^g^	–	–	–	72	72	80	80

^a^ST, MT, and LT stand for technologies in the short, medium, and long terms, respectively

^b^Sugarcane is processed during the sugarcane harvesting season (200 days/year)

^c^The recovery percentage relates to the amount of straw produced in the field. Baling for longer distances (50% of total area) and integral harvesting for short distances (50% of total area). Even with the increment in the sugarcane straw recovery percentages over time, higher projected biomass yields would allow maintaining the same amount of straw in the field (compared to the short term scenarios)

^d^Energy cane is processed only in the off-season (130 days), using the idle equipment used for conventional sugarcane during season, after some minor adjustments

^e^Energy cane is processed all-year round. The facility is designed to process both sugarcane and energy cane during the season (200 days). In the off-season (130 days), all installed capacity is employed to process only energy cane

^f^This yield is a result of process simulation based on the assumptions for 2G process in each time horizon. LCM refers to dry lignocellulosic material pretreated in the 2G process

^g^Produced biogas is purified and used to replace diesel limited to 70% in the agricultural operations and transport. Surplus biogas is burnt in internal combustion engines for electricity production

### Techno-economic and climate change assessment

The biomass production system was evaluated using the CanaSoft model, an in-house model that integrates and quantifies inputs and outputs in the biomass production stages (from preplanting operations to harvesting and transportation) within the VSB.

Mass and energy balances for each scenario were carried out using Aspen Plus® process simulation environment. These balances provided information for the estimation of operational costs and investments as well as for economic and environmental assessments.

Investments were based on VSB’s databank and methodology. In this approach, flows calculated through process simulation are used to estimate equipment capacities. In order to represent new engineering solutions and maturity evolution of 2G and biodigestion processes, reductions in medium- and long-term estimates equal to 10 and 20%, respectively, were considered for investments in these equipment. Economic assessment considered greenfield projects, i.e., new facilities. Main economic assumptions are presented in Table 2.

**Table 2:** Main financial parameters for economic assessment

Parameter	Value	Reference
Minimum acceptable rate of return (per year)	12%	Watanabe et al. [25]
Project life span (years)	25	Watanabe et al. [25]
Depreciation rate (linear, 10 years)	10%	Watanabe et al. [25]
Income taxes	34%	Milanez et al. [17]
Maintenance (%Capex)	3%	Milanez et al. [17]
Month/year of reference for economic parameters	July/2014^a^	–
Exchange rate (R$/US$)	2.30	Average of July, 2014
Enzyme cost—short term (US$/L 2G ethanol)	0.13	Estimate from suppliers
Enzyme cost—medium term (US$/L 2G ethanol)	0.08	Estimate from suppliers
Enzyme cost—long term (US$/L 2G ethanol)	0.06	Estimate from suppliers
Anhydrous ethanol price (R$/L)	1.34	Moving average (2004–2014) [26]
Electricity price (R$/MWh)	132.43	Average from auctions (2005–2013) [27]

^a^July/2014 was chosen based on the date when application of questionnaires and interviews with stakeholders took place

This study considered a vertically integrated model, i.e., a company controls both the agricultural and industrial production systems. In this sense, the biomass production cost in the agricultural phase, which is an output of the CanaSoft model, will be interpreted as the cost of biomass in the industrial cash flow analysis instead of considering sugarcane market prices [28]. This assumption is important because the management decisions regarding agricultural technologies to be used in the sugarcane field will impact the entire production chain, including the ethanol production costs at the industry.

#### Cost allocation

In this paper, the production cost was selected as the main economic result to compare both 1G and 2G ethanol competitiveness over time. The decision on production cost rather than other economic parameters, such as internal rate of return and net present value, was made in order to foster policy-making decisions focusing on reducing ethanol production costs.

The production cost is composed by two main components: operating and capital costs. Operating costs are associated with the annual expenses with feedstock, maintenance, labor, chemical substances, among other inputs; whereas the capital cost is calculated based on the annual payment that would be necessary to remunerate the total investment at an assumed 12% per year interest rate over a 25-year period. This amount of money represents the opportunity cost of the investment associated with the decision of building a new plant.

Considering that a biorefinery produces more than one product, an allocation criterion for operating and capital costs was applied. Ethanol production cost was calculated considering the allocation of overall yearly costs (operating and capital costs) between ethanol and electricity based on their participation on revenues. In the integrated 1G2G scenarios, an additional allocation step between 1G and 2G ethanol was performed, based on their participation in the ethanol output, to identify the impacts exclusively related to 2G ethanol [25]. In this case, for a same time horizon, it was considered that 1G ethanol has the same cost in both 1G and 1G2G scenarios. Therefore, all the additional costs are allocated to 2G ethanol.

#### Climate change impacts using life cycle assessment

The assessment of climate change impacts was performed through life cycle assessment (LCA) methodology. It is a broadly recognized methodology for estimating the environmental burden associated with a product, process, or activity, by the identification and quantification of energy and materials used and waste released, during its entire life cycle [25]. SimaPro software and the Ecoinvent database v2.2 were employed to obtain the datasets of the main inputs used in the product system evaluated (e.g., production of diesel, fertilizers, pesticides, and other chemicals used as input in the process) [25]. The scenarios were assessed using the climate change impact category from ReCiPe Midpoint H v 1.08 method [29], measured in g CO_2_ eq per MJ of ethanol. Equivalency factors of this category are based on the 100-year timeframe radiative forcing of a given greenhouse gas relative to carbon dioxide from IPCC 2007 report [30]. This impact category was selected because the potential to reduce GHG emissions has been one of the main motivations that drive research and use of renewable energy alternatives. For instance, targets of reductions in GHG emissions for biofuels replacing their fossil counterparts have been defined in the RSF2 regulatory framework [31].

## Results and discussion

### Techno-economic assessment

With expected technical improvements of both biomass production systems and industrial conversion, the biomass production costs and the products’ output change over time are shown in Tables 3 and 4, respectively.

**Table 3:** Amount and cost of biomass processed in each scenario

Scenario	Biomass processed (10^6^ t/year)^a^			Biomass production cost (US$/t)^a^		
	Sugarcane stalks	Sugarcane straw	Energy cane	Sugarcane stalks	Sugarcane straw	Energy cane
1G-base	2.00	–	–	28.00	–	–
1G-ST	4.00	0.25	–	29.11	27.09	–
1G-MT	4.00	0.34	1.72	20.31	20.47	14.15
1G-LT	4.00	0.39	4.17	16.15	20.66	12.10
1G2G-ST	4.00	0.25	–	29.30	27.09	–
1G2G-MT	4.00	0.34	1.72	20.73	20.47	14.33
1G2G-LT	4.00	0.39	4.17	16.68	20.66	12.63

^a^Amount and cost of sugarcane straw are expressed in dry basis. Values for sugarcane stalks and energy cane are expressed in wet basis

**Table 4:** Overall ethanol production (1G plus 2G), surplus electricity, and 2G yield for evaluated scenarios

Scenario	Ethanol production (10^3^ m^3^/year)	Surplus electricity (GWh/year)	Ethanol production (L/TC^a^)	Surplus electricity (kWh/TC^a^)	2G yield (L/t LCM^b^)
1G-base	170.4	–	85.2	–	–
1G-ST	339.7	697.5	84.9	174.3	–
1G-MT	438.3	1153.7	76.6	201.5	–
1G-LT	561.6	1769.8	68.8	216.7	–
1G2G-ST	433.9	274.3	108.4	68.6	237.5
1G2G-MT	667.4	403.2	116.6	70.4	293.0
1G2G-LT	989.1	555.4	121.1	68.0	348.9

^a^TC refers to metric tons of cane (either sugarcane stalks or energy cane)

^b^LCM refers to dry lignocellulosic material pretreated in the 2G process

Significant production cost reduction in sugarcane stalks and straw is achieved in long term (roundly 45 and 25%, respectively), mainly due to the increase in agricultural yields and use of biomethane as partial diesel replacement for agricultural mechanical operations. Cost increments due to lower density of the transported material when large amounts of straw are recovered and transported within the sugarcane stalks led to a small increase in straw costs for long-term scenarios. Therefore, besides the higher agricultural yields, straw costs are largely dependent on transportation costs. Further reduction in biomass production cost is observed with the introduction of energy cane, which is 25–30% lower than conventional sugarcane costs considering the same time horizon.

Specific ethanol production (per metric ton of cane) in 1G scenarios reduces over time due to the lower sugar content of energy cane in comparison to conventional sugarcane. In spite of that, the total annual ethanol production increases over time. This is a result of the larger amount of processed biomass, integration of 2G process and its technological advances.

The 2G yield for the different time horizons is a result of the assumed set of process parameters (detailed in Additional file 1) included as inputs in the mass and energy balances. In the short term, 237 L of ethanol is produced per metric ton of dry biomass processed in 2G unit. This figure is consistent with the information released by Raízen, one of Brazil’s pioneers in 2G ethanol production. Raízen claims to obtain 211 L per metric ton of dry biomass and expects to achieve 289 L at full capacity operation [12]. The theoretical ethanol yield for sugarcane biomass is estimated at about 422 L/t (dry basis) [32], so results for the long-term scenarios (~350 L/t) seem reasonable for 2030. The experience with 1G ethanol in Brazil has shown that economies of scale and technological advances may lead to a remarkable increase in its competitiveness as fuel [4].

Comparing 1G and 1G2G scenarios, 2G technology allows increasing ethanol production by 28, 52 and 76% in the short, medium, and long terms, respectively. The increase in ethanol production is a result of three main factors: increase in 2G yield; the introduction of energy cane; and reduction in steam demand (which increases biomass availability for 2G process) due to higher solid content and sugar concentration in the process steps. Energy cane presents higher fiber content, being a feedstock more suitable for 2G ethanol production. At the same time, it presents less readily fermentable sugars, thus the 1G ethanol yield is lower in energy cane in comparison to sugarcane, increasing the differences between 1G and 1G2G ethanol production for the same time horizon. For example, in the 1G2G-LT scenario, almost one billion liters of ethanol is produced per year, which is equivalent to an autonomous 1G plant processing around 12 million metric tons of conventional sugarcane per year. In the 1G2G scenarios, since all surplus lignocellulosic material is diverted to pretreatment, the electricity outputs are about one-third of those achieved in the 1G scenarios, considering the same time horizons.

The investment for each scenario is presented in Table 5. For comparison purposes, the investments were divided into two sectors: 1G+ interface and 2G. The first sector aggregates the processing areas usually found in the conventional first-generation ethanol plants (such as sugarcane reception, juice extraction, juice treatment, fermentation, distillation, CHP unit), biodigestion unit, administrative infrastructure, engineering, among others. The 2G sector includes areas specifically related to 2G processes, such as pretreatment, C5 fermentation, and enzymatic hydrolysis. The highest investment estimates are observed for the long-term scenarios (1G-LT and 1G2G-LT), due to the higher installed capacities and larger cost-intensive areas, such as the CHP (considering an optimized configuration) in 1G-LT and 2G plants (in particular, pretreatment and C5 liquor separation) in 1G2G-LT. At the same time, the lowest investment is related to 1G-base scenario, which has the lowest plant capacity and base technological level. The 1G+ interface investment in 1G2G scenarios is lower than those of 1G scenarios, even with the increase in fermentation and ethanol production areas that are shared between both processes, because the CHP unit capacity is smaller in 1G2G scenarios due to the use of biomass for 2G ethanol production.

**Table 5:** Estimated industrial investment for each scenario

Capex (US$ million)	1G-base	1G-ST	1G-MT	1G-LT	1G2G-ST	1G2G-MT	1G2G-LT
*1G* + *interface areas (subtotal)*	*153.3*	*436.4*	*472.8*	*627.6*	*410.2*	*455.6*	*586.4*
Administrative infrastructure, engineering, and utilities	41.6	99.3	105.8	137.7	127.1	131.4	163.7
Sugarcane reception, juice extraction, and evaporation	33.6	60.5	63.6	79.2	63.4	67.5	82.4
Ethanol production (fermentation, distillation/dehydration)	37.2	83.5	80.6	97.0	103.4	114.0	154.8
Combined heat and power (steam and electricity)	40.9	193.1	211.6	300.8	116.4	124.7	160.8
Biogas production, purification, and use for electricity generation (internal combustion engines)	–	–	11.1	13.0	–	18.0	24.7
*2G areas(subtotal)*	–	–	–	–	*184.9*	*163.4*	*190.1*
Pretreatment and C5 liquor separation/evaporation	–	–	–	–	106.5	102.3	127.7
C5 fermentation and yeast propagation	–	–	–	–	15.7	16.5	16.9
Enzymatic hydrolysis and C6 liquor separation	–	–	–	–	62.7	44.6	45.4
*Total*	*153.3*	*436.4*	*472.8*	*627.6*	*595.1*	*619.0*	*776.5*

**Fig. 2:**
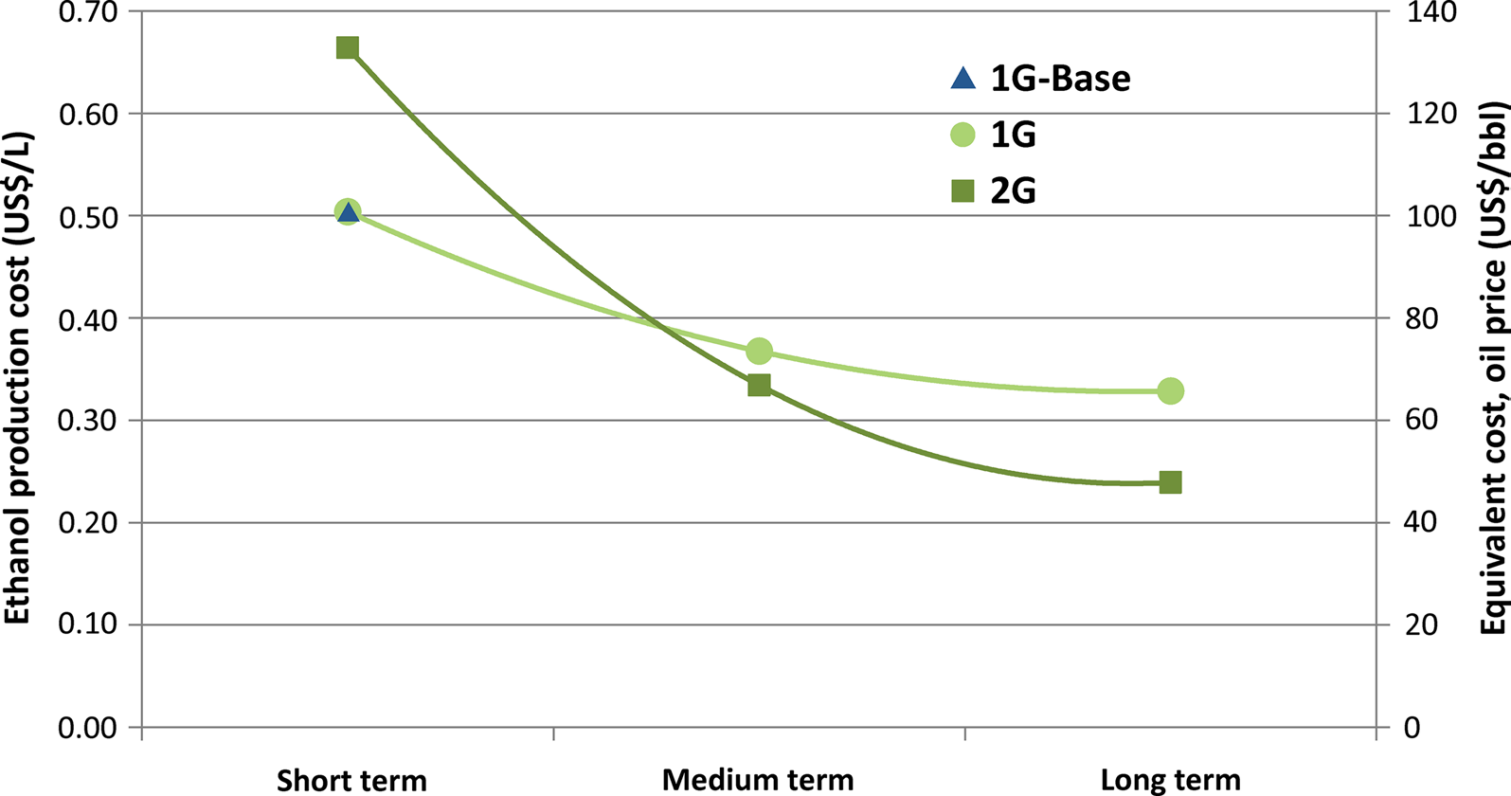
Projection of 1G and 2G ethanol production costs

**Fig. 3:**
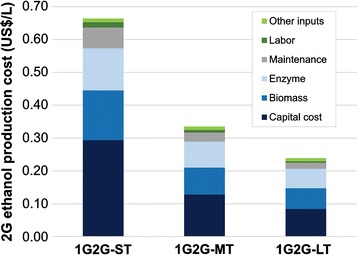
Breakdown of 2G ethanol production costs

Operational costs were estimated for each scenario based on the expenses with raw materials, labor, maintenance, enzymes, among others. These costs, added to the capital costs (relative to the investment), are allocated between the biorefinery products to obtain the ethanol production cost, as described in the “Methods” section. Projected ethanol production costs over time are shown in Fig. 2, along with the oil price required to produce gasoline with equivalent cost in energy basis. These estimations consider the share of West Texas intermediate (WTI) crude oil on the US gasoline price breakdown according to the data from the US Energy Information Administration [33]. Figure 3 shows the breakdown of 2G ethanol production costs for the three integrated 1G2G scenarios.

Ethanol production costs for 1G scenarios in the short term (1G-Base and 1G-ST) presented similar values. Although the modern configuration (1G-ST) has an extra revenue from electricity (which reduces the fraction of the total costs allocated to ethanol), higher capital costs (mainly related to CHP unit) and additional costs with straw recovery result in increased ethanol production cost. 2G ethanol presents higher costs than 1G in the short term mainly due to high capital cost associated with the additional investment and enzyme costs that together represent roughly 65% of the total 2G cost. Although both costs are expected to reduce over time, the trend shows that 2G ethanol cost will be lower in the medium and long terms if the expected technological advances are obtained. For instance, the capital cost, which is the main component of production cost, is significantly reduced due to lower residence times and higher solid contents in the 2G process. Additionally, reductions in investment estimates in the medium and long terms are anticipated because of the development of local equipment manufacturers, which are assumed to deliver more cost-competitive solutions overtime. Biomass costs also decrease over time as a consequence of projected developments in the agricultural production system, including the introduction of energy cane. Another driver for reduction in 2G ethanol production costs is the cost of enzyme, which is expected to have lower contribution with the development of more efficient enzymatic cocktails and establishment of local enzyme producers.

As Figs. 2 and 3 depicted, 2G cost is lower than 1G from medium to long terms due to the relatively faster decrease of 2G costs. These results rely on a variety of assumptions. First, the 2G technology is still in the beginning of its technological learning curve whereas 1G is a mature technology which has a lower potential for cost decrease in the industrial stage. Biomass cost reductions are still possible (due to new sugarcane varieties and the introduction of energy cane); therefore, 1G ethanol can experience further cost reduction mostly related to advances in the agricultural production systems. Second, the high costs associated with 2G technology rely on the current choices of industrial routes and equipment design (such as those dedicated to pretreatment area) that may evolve over the years. Considering the potential of reduction in capital, and enzyme and biomass costs with the increase of industrial yields, 2G technology has higher potential of cost reduction over time.

The comparison of ethanol production costs and the oil price in the international scenario (Fig. 2) indicates that both 1G and 2G ethanol are competitive in the short term if oil prices exceed US$ 100/bbl and US$ 130/bbl, respectively. In the long term, ethanol competitiveness is achieved for oil prices above US$ 65/bbl and US$ 45/bbl for 1G and 2G ethanol, respectively. For comparison, although oil price has recently dropped to lower levels, it ranged from US$ 60/bbl to US$ 115/bbl between 2010 and 2014 [34].

### Sensitivity analysis

**Fig. 4:**
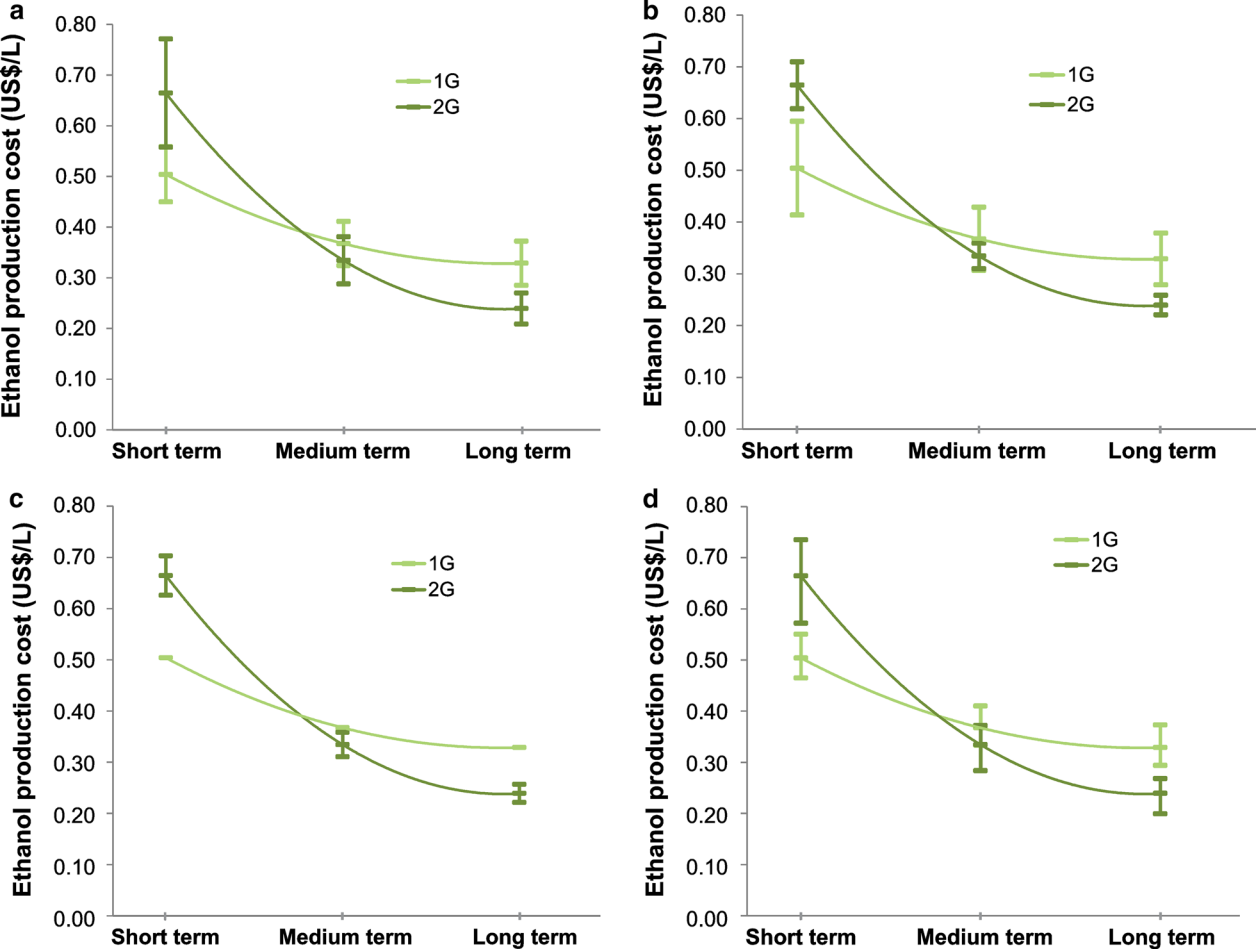
Sensitivity analysis for ethanol production cost. Ethanol production cost considering variations in **a** capital cost (±30%), **b** biomass cost (±30%), **c** enzyme cost (±30%), **d** electricity price (±50%)

Sensitivity analysis was carried out to evaluate the impact of possible variations on biomass, enzymes, and capital costs in both 1G and 2G ethanol production costs. In addition, variation in electricity price, which affects the allocation of production costs, was applied to show its influence on the results. Figure 4 presents the projection curve of ethanol production cost including a sensitivity analysis.

Due to the larger investment of integrated 1G2G scenarios, capital cost has a higher impact on 2G ethanol production cost, especially in the short term, overlapping with 1G ethanol production cost. On the other hand, biomass influences more 1G ethanol, since it is the most important component of its production cost. As enzyme affects only 2G ethanol production cost, a lower impact in the projection curve is observed. Even so, the comparison shows that the uncertainties in enzyme price could approximate 1G and 2G ethanol production costs in the medium term.

The impact of electricity price on the ethanol production cost is on allocation. For higher prices, electricity participation in the revenues increases, reducing 1G ethanol production cost. Similarly, 1G2G ethanol production cost also decreases but by a smaller factor (due to lower electricity output). However, because of the reduction in the 1G ethanol production cost, 2G ethanol production cost increases. In this case, a variation of 50% was assumed due to the high uncertainty and variability of electricity prices in Brazil. The impact on hydropower availability affects reference prices of other renewable sources in the electricity market—such as solar, wind, and biomass—mainly due to the increasing demand of high cost electricity dispatched to the grid (from oil and natural gas-fired power plants).

It is worth highlighting that the exchange rate is also an important variable, which impacts 2G ethanol production costs in the Brazilian currency. Considering that a significant share of imported 2G equipment (pretreatment reactor, for instance) is estimated in US dollars and that enzymes are reliant on prices set by foreign companies, a high exchange rate volatility may increase uncertainties to the 2G production costs in the Brazilian market, especially in the short term. However, as much as the 2G ethanol internal market becomes more competitive and mature over time, it is expected that this effect may decrease due to a possible expansion of local 2G equipment and enzyme manufacturers.

### Climate change impacts

**Fig. 5:**
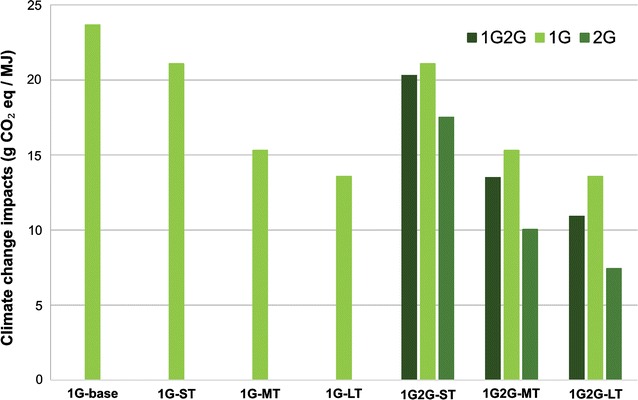
Climate change impacts per unit of energy of ethanol produced in the considered scenarios

Environmental impacts for electricity, 1G ethanol, and 2G ethanol were allocated using the same criteria employed in the economic assessment; thus, impacts are proportional to the participation of each product on revenues. Figure 5 presents climate change impacts of ethanol for the evaluated scenarios, considering a cradle-to-gate analysis. At this point, for comparison purposes, impacts related to biofuel distribution and use were not included since there is no expected difference in these life cycle steps for the considered scenarios.

The highest environmental impacts (around 24 gCO_2_ eq/MJ) are related to the 1G-base scenario, which commercializes only ethanol. In the 1G-ST, part of the impacts is allocated to electricity production, therefore reducing ethanol impacts. Increase in biomass yield and use of biomethane as diesel replacement play an important role in the reduction in GHG emissions, dropping it below 14 gCO_2_ eq/MJ in the 1G-LT scenario. Due to higher ethanol yields (i.e., more ethanol is produced with the same biomass), the climate change impacts for 2G ethanol production were lower than that of 1G scenarios for all time horizons. The expected advances on 2G technology led to a substantial climate change impact mitigation, resulting in values as low as 7.5 gCO_2_ eq/MJ in the 1G2G-LT scenario.

To compare ethanol GHG emissions with those from gasoline, the entire life cycle needs to be considered (cradle-to-grave analysis). In this sense, the emissions from ethanol distribution and use (1.03 g CO_2_ eq/MJ, for all the evaluated scenarios) were added to ethanol cradle-to-gate results from Fig. 5. The LCA was applied to both ethanol and gasoline under same conditions, methods, and databases, making this comparison reasonable. Considering that the production and use of gasoline in Brazil emits 87.4 g CO_2_ eq/MJ, 1G ethanol in medium and long terms and 2G ethanol present climate change impact reduction by over 80% in comparison to gasoline. This result is in line with other important studies for Brazilian sugarcane ethanol (not including land use change emissions or ethanol transportation to other countries) [35, 36]. These climate change impacts allow ethanol to be classified as an advanced biofuel according to RSF2 regulatory framework [31].

### Public policies

There is a clear potential for 2G ethanol to achieve a lower production cost compared to 1G ethanol in the medium to long term, even considering several technological and market uncertainties. However, 2G process is at the beginning of its learning curve and expected improvements will depend on the diffusion and adoption of these new technologies. Therefore, additional mechanisms to encourage R&D, as well as production and consumption of 2G ethanol in Brazil, can be proposed to accelerate the path along the learning curve.

By stimulating investment in the new 2G plants, there would be greater incentive for the development of the entire production chain, such as the establishment of local equipment and enzyme suppliers. Some incentives include premium prices, mandates, specific auctions, taxes exemption, and special credit lines for applied R&D focused on the main drivers of 2G ethanol production costs. For instance, specific funding program for the development and use of energy cane could significantly reduce biomass cost and, integrated to 2G technology, would considerably increase ethanol production per crop area.

Therefore, if the suggested mechanisms are successfully implemented, they are likely to play an essential role to rapidly reach larger gains and, then, accelerate the diffusion of a new paradigm in the sugarcane industry, increasing the competitiveness of the sector [17].

## Conclusions

Improvements in the biomass production system were projected considering the increase in agricultural yields, use of biomethane as diesel replacement, and introduction of energy cane beyond other expected improvements. Reduction in biomass costs reached about 55% when comparing energy cane in the long term to conventional sugarcane in the short term, for example.

The integration of 2G technology allowed an increase in ethanol production by 28, 52 and 76% in the short, medium, and long terms, respectively. These increments are mainly associated with the advances on 2G technology and the processing of energy cane.

In terms of ethanol production costs, although 2G ethanol presents higher cost in the short term, the trend is that 2G ethanol cost will be competitive in the future. Therefore, public policies specifically designed to motivate the production and consumption of 2G ethanol in Brazil are essential to flatten the learning curve of 2G technology.

Environmental assessment results showed that both 1G and 2G ethanol are able to mitigate climate change impacts in comparison to gasoline, but higher benefits are achieved with 2G ethanol production. These results are aligned with the commitment of the Brazilian Government in its Intended Nationally Determined Contribution (INDC) to COP 21—Paris, 2015—to reduce GHG emission by increasing the share of sustainable biofuels in the Brazilian energy matrix [37].

## Additional file

**Additional file 1.** Detailed information about biomass composition and production system as well as industrial process for different technology levels and timeframes.

## Abbreviations

1G: first generation
1G2G: first and second generation
2G: second generation
C5: 5-carbon molecules
C6: 6-carbon molecules
Capex: capital expenditure
CHP: cogeneration of heat and power
CTBE: Brazilian Bioethanol Science and Technology Laboratory
GHG: greenhouse gas
LCA: life cycle assessment
LCM: dry lignocellulosic material pretreated in the 2G process
LT: long term (2026–2030)
MT: medium term (2021–2025)
Mtoe: million metric tons of oil equivalent
R&D: research and development
RFS: renewable Fuel Standard
ST: short term (2016–2020)
TC: metric tons of cane
VSB: Virtual Sugarcane Biorefinery
WTI: West Texas intermediate
