# Supplementary material for: Techno-economic analysis and climate change impacts of sugarcane biorefineries considering different time horizons
Source: Biotechnol Biofuels. 2017 Mar 14;10:50. doi: 10.1186/s13068-017-0722-3 (PMC5348788; doi:10.1186/s13068-017-0722-3)
Supplement: Supplementary file 1 — Additional file 1. Detailed information about biomass composition and production system as well as industrial process for different technology levels and timeframes. [file 13068_2017_722_MOESM1_ESM.pdf]

Additional file 1. Detailed information about biomass composition and production system as well as industrial process for different technology levels and timeframes.

Title: Techno-economic analysis and climate change impacts of sugarcane biorefineries considering different time horizons

Tassia L. Junqueira[a1], Mateus F. Chagas[a,b], Vera L. R. Gouveia[a], Mylene C. A. F. Rezende[a], Marcos D. B. Watanabe[a], Charles D. F. Jesus[a], Otavio Cavalett[a], Artur Y. Milanez[c], Antonio Bonomi[a,b]

[a] Laboratório Nacional de Ciência e Tecnologia do Bioetanol (CTBE), Centro Nacional de Pesquisa em Energia e Materiais (CNPEM), Caixa Postal 6192, CEP 13083-970, Campinas, São Paulo, Brazil

[b] Faculdade de Engenharia Química, Universidade Estadual de Campinas (UNICAMP), Campinas, São Paulo, Brazil

[c] Departamento de Biocombustíveis, Banco Nacional de Desenvolvimento Econômico e Social (BNDES), Rio de Janeiro, Brazil

[1] Corresponding author: tassia.junqueira@bioetanol.org.br

Table A.1. Energy cane and sugarcane composition and productivity (adapted from Milanez et al., 2015).

**Composition[a] (% wt)**	**Sugarcane[b]**	**Sugarcane Straw**	**Energy cane[d]**
Water	70.3	15.0[c]	66.8
Fibers	12.7	77.9	21.3
Sucrose	14.0	4.3	8.1
Reducing sugars	0.6	0.2	2.5
Others	2.4	2.6	1.3

[a] Does not include mineral impurities.

[b] Considering only stalk composition.

[c] Moisture for straw recovered through baling. For integral harvesting, moisture varies with the amount recovered.

[d] Considering full composition (stalk + straw).

Table A.2. Assumptions for biomass production system (adapted from Milanez et al., 2015).

**Scenarios**	**Units**	**1G-Base**	**Short term**	**Medium term**		**Long term**	
Cane (SC or EC)[a]		SC	SC	SC	EC	SC	EC
Milling capacity	Mt/y	2	4	4	1.72	4	4.17
Agricultural yield	t/ha.y	80	80	100	200	120	250
Transport distance	km	25	35	35	35	35	35
Longevity	harvest/cycle	5	5	5	10	5	10
Reduced tillage and precision agriculture	% of total area	20	20	80	80	100	100
Planting system	% Manual	40	20	-	-	-	-
	% Mechanized	60	80	100	100	-	-
	% ETC[b]	-	-	-	-	100	100
Harvesting system	% Manual	30	10	-	-	-	-
	% Mechanized	70	90	100	100	-	-
	% ETC	-	-	-	-	100	100
Straw recovery[c]	%	-	50	60	100	70	100
Diesel replacement[d]	%	-	-	70	70	70	70

[a] SC means sugarcane and EC, energy cane;

[b] ETC means Controlled Traffic Structure, a new machinery for sugarcane planting and harvesting under development at CTBE.

[c] Percentage in relation to the amount of straw produced in the field. Recovery method is baling for longer distances (50 % of total area) and integral harvesting for short distances (50 % of total area);

[d] Limit percentage of diesel replacement by biomethane (for use in machinery and trucks). Surplus biogas is used as fuel in internal combustion engines for electricity production.

Table A.3. Main operating conditions and yields for 1G ethanol production, cogeneration and biodigestion technologies (adapted from Milanez et al., 2015).

Parameter	Base	Short term	Medium term	Long term
**1G process and CHP unit**				
Sugars extraction efficiency (%)				
Sugarcane	96.0	95.4[a]	95.4[a]	95.4[a]
Energy cane (crushed by sugarcane equipment during offseason)	-	-	90	90
Energy cane (crushed by a dedicated simpler equipment)	-	-	80	80
Ethanol titer in wine (g/L)	70	70	85	85
Fermentation yield (%) – sugarcane juice	90	90	90	90
Dehydration process	Azeotropic distillation	Molecular sieves	Molecular sieves	Molecular sieves
Reduction in steam consumption due to heat integration (%)	-	10	20	30
Boiler pressure (bar)	22	65	65	65
Boiler efficiency based on the LHV[b] (%)	75.0	87.8	87.8	87.8
Turbine efficiency (%)	70.6	83.3	83.3	83.3
**Biogas production and utilization**				
Chemical oxygen demand (kg COD/m^3 vinasse)	-	-	21	21
COD removal efficiency	-	-	72	80
CH_4 production (Nm^3/kg COD removed)	-	-	0.29	0.31
Efficiency of internal combustion engine (%)	-	-	38	38

[a] Extraction efficiency is affected by the larger amount of fibers due to integral harvesting.

[b] LHV – lower heating value

Table A.4. Main operating conditions and yields for 2G ethanol production technologies (adapted from Milanez et al., 2015).

**Parameter**	**Short term**	**Medium term**	**Long term**
**Pretreatment**			
Type	Steam explosion	Steam explosion	Steam explosion
Temperature (°C)	190	200	210
Residence time (min)	15	10	5
Solids content (%)	*defined by the steam required to achieve reactor temperature*		
Cellulose solubilization (%)	5	5.5	5.5
Xylan conversion to xylose (%)	30	45	60
Xylan conversion to xylose oligomers (%)	30	25	20
Xylan degradation to furfural (%)	10	10	10
Lignin solubilization (%)	10	10	10
Acetyl group conversion to acetic acid (%)	70	80	90
**Enzymatic Hydrolysis**			
Temperature (°C)	50	50	65
Pressure (bar)	1.0	1.0	1.0
Residence time (h)	48	36	36
Solids content (%)	15	20	25
Cellulose conversion to glucose (%)	60	70	80
Xylan conversion to xylose (%)	60	70	80
**Deoligomerization and C5 fermentation**			
Temperature (°C)	33	33	33
Residence time (h)	48	36	24
Oligomers conversion to monomeric sugars (%)	80	90	90
C6 conversion to ethanol (%)	90	90	90
C5 conversion to ethanol (%)	80	80	85
Cell Recycling (%)	80	90	95
**C6/C12 fermentation**			
Operational conditions	same as 1G	same as 1G	same as 1G
C6/C12 conversion to ethanol (%)	88	90	90

Reference

Milanez, A. Y., Nyko, D., Valente, M. S., Sousa, L. C., Bonomi, A., Jesus, C. D. F., Watanabe, M. D. B, Chagas, M. F., Rezende, M. C. A. F., Cavalett, O., Junqueira, T. L., Gouveia, V. L. R., 2015. De promessa a realidade: como o etanol celulósico pode revolucionar a indústria da cana-de-açúcar: uma avaliação do potencial competitivo e sugestões de política pública. BNDES Setorial, 41, 237-294.
